# Application evaluation of intraoperative ultrasound combined with neuro electrophysiological detection in the spinal cord glioma surgery

**DOI:** 10.12669/pjms.37.3.3638

**Published:** 2021

**Authors:** Xin Li, Zhen-jie Liu, Liang Liang, Hai-qing Dong

**Affiliations:** 1 Xin Li, Department of Neurosurgery, Baoding No.1 Central Hospital, Baoding, 071000, Hebei, China; 2 Zhen-jie Liu, Department of Neurosurgery, Baoding No.1 Central Hospital, Baoding, 071000, Hebei, China; 3 Liang Liang, Department of Neurosurgery, Baoding No.1 Central Hospital, Baoding, 071000, Hebei, China; 4 Hai-qing Dong, Department of Neurosurgery, Baoding No.1 Central Hospital, Baoding, 071000, Hebei, China

**Keywords:** Intraoperative ultrasound, Neuro electrophysiological detection, Spinal cord glioma, Traditional microsurgery

## Abstract

**Objective:**

To observe application values of intraoperative ultrasound combined with neuro electrophysiological detection in the spinal cord glioma surgery.

**Methods:**

Sixty patients with spinal cord glioma hospitalized in Baoding First Central Hospital from January 2016 to January 2018 were selected, randomly divided into two groups by the random number table method, with 30 cases of each group. PASS software was used to calculate the sample size. The control group was treated with traditional microsurgery, while the experimental group was treated with intraoperative ultrasound combined with neuro electrophysiological testing. The operation time, intraoperative blood loss, postoperative hospital stays, degree of tumor resection, clinical efficacy, recovery of neurological function, recovery of health status, quality of life score, and 2-year recurrence rate of the two groups of patients were observed and compared.

**Results:**

The operation time of the experimental group was longer than that of the control group, and the postoperative hospital stay was shorter than that of the control group. The complete tumor resection rate, complete remission rate and postoperative scale scores of the experimental group were significantly higher than those of the control group, while the recurrence rate within two years was significantly lower than that of the control group. The above differences were statistically significant (p<0.05).

**Conclusions:**

Intraoperative ultrasound combined with neuro-electrophysiological detection for spinal glioma has more adequate protection of nerve function, high clinical complete remission rate, more thorough tumor resection, and lower recurrence rate than traditional microsurgery, which is worthy of clinical application.

## INTRODUCTION

Spinal cord glioma is a rare diffuse midline glioma.^1^ The average age of patients attacked by it is 40; and a ratio of male to female cases is 18:7. Predilection sites are proved to be cervical and thoracic segments, while its incidence in lumbar vertebra is low.^2^ At present, the major treatment approach of this disease is surgery.^3^ However, performing an operation may damage the normal spinal cord and relevant nerves because the tumor is in the spinal cord, causing corresponding postoperative neurological disorders among patients. If the mass excision level is excessively low for the purpose of protecting nerves of the normal spinal cord, it is much likely that corresponding patients suffer a rather early relapse after the surgery. For this reason, it is of particular importance to carry out intra-operative neuro electrophysiological monitoring to avoid nerve injuries during the surgery.^4^

In the present study, neuro electrophysiological detection is combined with an intraoperative ultrasonic probe, effectively preventing nerve injuries on one hand and presenting explicit guidance on the mass excision extension. At last, an obvious effect is achieved. The details are now reported as follows.

## METHODS

### Ethical approval:

The study was approved by the Institutional Ethics Committee of Baoding First Central Hospital (No# [2020]042; Date: 21 May, 2020), and written informed consent was obtained from all participants.

### Inclusion criteria:

1. Patients with typical neurological symptoms and signs.
2. Patients with spinal cord glioma as suggested by preoperative MRI.^5^
3. Patients diagnosed with glioma according to postoperative pathologic findings.
4. Patients willing to participate in the study and sign the informed consent.
5. Patients cooperating with this research and showing no obvious mental disorders.

### Exclusion criteria:

1. Patients whose preoperative examination or postoperative pathology suggests non-glioma.
2. Patients with severe mental disorders, failing to cooperate in this research.
3. Patients with other severe underlying diseases that cannot be corrected and intolerant to surgery.

PASS software was used to calculate the sample size. Sixty spinal cord glioma patients hospitalized in Baoding First Central Hospital from January 2016 to January 2018 were selected. According to the principle of random control, these 60 patients were randomly divided into two groups by the random number table method, with 30 cases in each group. When performing tumor resection, the control group was treated with traditional neurosurgery microscopy techniques, while the experimental group was treated with intraoperative ultrasound combined with neuro electrophysiological monitoring techniques. There is no significant difference in the general information of the two groups of patients (p>0.05), and the groups are comparable (Table-I).

**Table-I:** Comparative analysis on general data of patients in experimental and control groups ( ±S) n=30

Indexes	Experimental group	Control group	t/χ^2^	p
Male (number, %)	17(57%)	19(63%)	0.28	0.60
Age	41.50±9.07	42.37±8.83	0.38	0.71
***Tumor site***				
Cervical segment	12	10	0.29	0.59
Thoracic segment	14	15	0.07	0.79
Lumbar segment	4	5	0.13	0.72
KPS score	68.54±12.27	69.29±13.10	0.23	0.82
SF-36 score	47.74±3.35	49.13±5.24	1.22	0.23

p>0.05.

### Surgical methods

### Traditional microsurgery:

Patients in the control group need to suffer general anesthesia, posed at a lateral position or lateral prone posture. The posterior median surgical approach was adopted, and the tissue was opened layer by layer to fully expose the tumor, and the tumor boundary was determined according to the preoperative MRI image. Then, the mass was identified under a microscope, separated step by step from the anterior pole to the posterior pole and excised. Under the circumstance of a failure in determining the tumor boundary, intracapsular resection should be performed or the excision should be as close to the tumor side as possible, reducing spinal cord injuries. Moreover, feeding vessels of the spinal cord should be also preserved to the greatest extent. Subsequent to tumor excision, pia mater spinalis, arachnoid and endorhachis were sutured in an interrupted way. An epidural drainage tube was placed; the site where the involved vertebral plate was resected should be repaired with a titanium alloy sheet and subjected to spinal stabilization. After that, incisions were sutured layer by layer and the excised tumor was submitted for pathological examinations as routine.

### Intraoperative ultrasound combined with neuro electrophysiological monitoring:

In the experimental group, a method of intraoperative ultrasound combined with neuro electrophysiological monitoring is adopted. Here, the required operating equipment includes Oxford 10-channel intramedullary neuro electrophysiological monitor (Medelec Synergy, UK), a Sonosite 180 color doppler (America) and an ultrasonic probe applied during the surgery. After general anesthesia of patients, an electrode was placed to record somatosensory evoked potential (SEP) waveforms of patients and set up the corresponding baseline. Incisions are made by a posterior midline approach. Then, endorhachis was exposed, and the intraoperative ultrasonic probe was utilized to define the tumor site and size, and identify the tumor boundary. In line with ultrasound results, endorhachis was cut open, so was the spinal cord longitudinally. Therefore, relationships of the mass and the normal spinal cord were observed under a microscope. Separation of the mass started at a site where the most obvious boundary is found between the mass and the spinal cord. The operation should be suspended if the amplitude suddenly drops or the waveform disappears, then local rinsing was performed with warm saline. The separation of mass cannot proceed until the waveform is regained. Moreover, ventral lesion excision in the spinal cord should be combined with spontaneous electromyogram and motor evoked potential (MEP) detection. During the excision, D waves were observed at the time of cutting open and separating the tumor, making sure that its amplitude remained at 50% and above. In this way, impairment of motor functions may be avoided. Since the surgery was completed, endorhachis was sutured, a drainage tube placed extradurally, and incisions sutured layer by layer after vertebral plate reduction fixation.

### Observation index:

Surgery related indexes: Differences in patients’ indexes were observed, including their time of operation, intraoperative blood loss, postoperative LOS, tumor excision levels and clinical efficacy. Furthermore, tumor excision levels were evaluated by postoperative MRI, comparing their differences between the groups.

The tumor excision levels can be evaluated based on the following methods. In terms of complete excision, the tumor can be completely excised if its boundary is observed to be clear. After the surgery, no spinal cord abnormality enhancement areas are found by postoperative MRI. As for subtotal resection, most tumors are excised as their boundaries are proved to be vague under a microscope, and postoperative MRI suggests residual tumors take a proportion of less than 5%. Concerning partial excision, some tumors are excised in a palliative manner due to a blur tumor boundary and an unclear boundary between the normal spinal cord and it. As indicated by postoperative MRI, over 5% of the tumors are left behind.^6^

In conformity with therapeutic response evaluation standards of the spinal cord glioma,^7^ clinical efficacy can be classified into four grades of complete remission (CR), partial remission (PR), stabilization (SD) and progression (PD). On this basis, differences in clinical efficacy of both groups are comparatively analyzed.

### Follow-up visit related indexes:

All patients were followed up for two years. One year after surgery, neural functional recovery scores (ADL rating scale), health condition recovery scores (KPS rating scale) and life quality scores (SF-36 scale) of patients in both groups are respectively recorded for comparative analysis on their recovery status. Take two years postoperatively as the observation endpoint, record and compare the recurrence rate of the two groups of patients.

### Statistical analysis:

While statistics about data is made by software SPSS 20.0, relevant measurement data are denoted as (χ̄ ±S). Also, an independent sample T-test is carried out for data analysis between experimental and control groups, comparison of recurrence rates is fulfilled through χ^2^ test. In case of P<0.05, it indicates that their differences are of statistical significance.

## RESULTS

As shown by comparison results, the time of operation in the experimental group is longer than that in the control group and their differences are statistically significant (p=0.02). As for postoperative LOS, it is shorter in the experimental group by contrast to the control group. Likewise, differences in it are significant (p=0.02). However, no significant differences lie in intraoperative blood losses between the groups (p=0.60), as shown in Table-II.

**Table-II:** Comparative analysis on surgical conditions (χ̄ ±S) n=30

Groups	Time of operation (min)*	Blood loss (ml) Δ	Postoperative LOS (d)*
Experimental	224.34±35.26	75.31±14.42	16.58±4.73
Control	203.76±33.17	77.35±15.48	19.97±6.41
t	2.33	0.53	2.33
p	0.02	0.60	0.02

* p<0.05; Δp>0.05.

The rate of complete tumor removal in the experimental group was significantly higher than that in the control group (p=0.02). The complete remission rate of the experimental group was significantly higher than that of the control group (p=0.04), as shown in Table-III.

**Table-III:** Comparative analysis on surgical results related indexes (χ̄ ±S) n=30

Indexes	Experimental group	Control group	χ^2^	P
***Excision level***				
Complete excision*	20	12	4.28	0.02
Subtotal resection*	7	14	3.58	0.04
Partial excision	3	4	0.16	0.68
Complete+ Subtotal resection	27	26	0.16	0.68
***Clinical effects***				
CR*	16	10	4.44	0.04
PR*	8	15	5.52	0.01
SD	5	3	0.58	0.44
PD	1	2	0.35	0.56
CR+PR	24	25	0.11	0.74

***Notes:*** ***CR:*** Complete remission, ***PR:*** Partial remission ***SD:*** Stabilization, ***PD:*** Progression,

* p<0.05

The ADL score, KPS score, and SF-36 quality of life score of the two groups of patients at one year after surgery were improved compared with those before treatment (p=0.00). The experimental group is significantly better than the control group, and the score is significantly higher than the control group (ADL & KPS, p=0.00; SF-36, p=0.01), as presented in Table-IV.

**Table-IV:** Comparative analysis on postoperative function scores (χ̄ ±S) n=30

Observation targets	ADL scores				KPS scores				SF-36 life quality scores			

Groups	Before treatment*	After treatmentΔ	t	p	Before treatment*	After treatmentΔ	t	p	Before treatment*	After treatmentΔ	t	p
Experimental Δ	45.43±5.34	76.97±6.54	20.46	0.00	68.54±12.27	92.61±12.47	7.54	0.00	47.74±3.35	80.37±10.24	16.58	0.00
Control Δ	43.73±4.60	60.23±4.72	13.71	0.00	69.29±13.10	82.25±14.33	3.66	0.01	49.13±5.24	73.72±8.48	13.51	0.00
t	1.32	11.37			0.23	2.98			1.22	2.74		
p	0.19	0.00			0.82	0.00			0.22	0.01		

* p>0.05, Δp<0.05

**Table-V:** Postoperative recurrence rate comparison (χ̄ ±S) n=30

Indexes	Number of relapses	Recurrence rate (%)*
Experimental group	4	13.33
Control group	11	36.67
χ^2^		4.36
p	0.04

* p<0.05

Within two years after surgery, four cases (13.33%) in the experimental group recurred, and 11 cases (36.67%) in the control group recurred. There was a significant difference in the recurrence rate between the two groups (p=0.04).

## DISCUSSIONS

Among central nervous system diseases, spinal cord glioma has a comparatively low incidence rate and takes about 10% in all tumors of spinal canal. Moreover, its annual incidence rate can be expressed as 0.22 per 100,000 people.^8^ According to pathological patterns, there are ependymoma, astrocytoma, ganglioglioma, ganglion neurocytoma, mixture astrocytoma and spongioblastoma primarily. While adults are mostly attacked by ependymoma, astrocytoma frequently occurs in children.^9^ Now, therapeutic regimens for spinal cord glioma mainly include microsurgery^10^, radiotherapy^11^ and immunotherapy.^12^

Spinal cord glioma is segmentally distributed; and the number of male patients is above that of female patients, occupying a proportion of about 57%.^13^ As far as diagnosis is concerned, applications of MR are featured with a rather high diagnostic value. Through MR, tumor sites and morphologies of tumors can be observed three-dimensionally. In addition, their sizes, quantities and neighboring relationships with the spinal cord are all clarified; moreover, the nature of some tumor may be event identified. Regarding its specificity and sensitivity, they are calculated to be at least 88.2% and 95% respectively.^14^

It is now deemed^15^ that aggressive surgical excision is the most effective therapeutic strategy for most spinal cord gliomas. With constant advances in imaging techniques and increasingly clear operating microscopes, an increasing number of tumors can be detected at an early stage. In opinions of some scholars, a tumor should be excised as soon as possible to the greatest extent once it is found, protecting spinal cord functions of patients from further damages.^16^ It is unanimously believed that intramedullary tumor excision by microsurgery is the most effective method that can be taken to protect normal spinal cord functions. Ideally, while the greatest tumor excision level is achieved, damages to the spinal cord are minimized simultaneously.^17^ With the development of neuro electrophysiological detection technology and intraoperative ultrasound technology, this ideal can be realized. In a 2.7-year follow-up study, with the aid of neuro electrophysiological monitoring during glioma surgery, the patient recovered well, and the preoperative symptoms, such as numbness and pain of the limbs, were significantly reduced. There is no obvious motor dysfunction after operation, and there is almost no recurrence after operation.^18^

This study confirmed that the experimental group used intraoperative electrophysiological testing, and the postoperative hospital stay was significantly shorter than that of the control group. Combination with intraoperative neuro electrophysiological monitoring plays a critical role in excision of intramedullary spinal cord tumors. Especially in the context where anatomic landmarks or tumor boundaries are unclear or cannot be identified, the surgery may be guided by continuous monitoring SEP.^19^ Once SEP fluctuates or disappears at the time of tumor cut-off or separation, the surgery should be immediately suspended and the site rinsed with warm saline. In most cases, such a situation may become normal again naturally. However, SEP, featured with low sensitivity but a high false-positive rate,^20^ only reveals sensory pathways of the spinal cord. If SEP can be combined with MEP monitoring during the surgery, such a defect can be settled.^21^ In this study, it is demonstrated that as patients in the experimental group undergo intraoperative neuro electrophysiological detection, their postoperative LOS is apparently shorter than that in the control group. The neural functional recovery scores, health condition recovery scores and life quality scores one year after the surgery were all improved to a greater extent in the experimental group than those in the control group. This proves that tumor excision assisted by intraoperative neuro electrophysiological detection shows great advantages in nerve preservation and postoperative neural functional recovery.

Application of ultrasonic probe in the surgery has an important guidance meaning in determining the excision range. Moreover, intraoperative ultrasound has some superiorities of being non-invasive, easy to use and highly accurate, etc. For this reason, ultrasound is required for accurate positioning in each link of the surgery, including vertebral plate excision range, length of dura mater that is cut off^22^ and length of the spinal cord that is cut off. It turns out that intraoperative ultrasonic imaging is highly consistent with postoperative MRI examinations.^23^ As suggested in the present research, the complete excision rate of the experimental group is apparently higher than that of the control group for a reason that real-time monitoring is achieved through intraoperative ultrasound for the former. In terms of the complete remission rate, the experimental group also outperforms the control group. Additionally, the tumors can be excised more completely in the experimental group. The two-year recurrence rate is significantly different between the two groups, which is pointed out that intraoperative ultrasound based real-time monitoring has some obvious advantages.

### Limitations and Recommendations of the study:

In addition to retrospective analysis and a small sample size, the spinal cord glioma is only analyzed and investigated from the perspective of surgical methods, failing to differentiate different pathologic types of the tumor during the research. In addition, follow-up visits and analyses are all performed based on types of patients, covering the excision rate and postoperative recurrence rate of ependymoma and astrocytoma that are subjected to intraoperative ultrasound combined with neuro electrophysiological detection.

## CONCLUSIONS

To sum up, although the combination of intraoperative ultrasound with neuro electrophysiological detection, in comparison with the traditional microsurgery, produces a quite complicated operating process and requires a longer time of operation. Patients can benefit a lot from it, such as a short postoperative LOS, more sufficient protection over neurological functions during the surgery, a high clinical complete remission rate, obvious neural functional recovery and significant life quality improvement. Thanks to ultrasonic detection, the tumor can be excised more completely. Ultrasound combined with neuro electrophysiological detection is of great significance in improving the quality and safety of spinal glioma surgery, and it is worthy of clinical application.

### Authors’ Contributions:

**XL and ZL** designed this study and prepared this manuscript, and are responsible and accountable for the accuracy or integrity of the work.

**LL** collected and analyzed clinical data.

**HD** significantly revised this manuscript.
